# Genome Sequence of a Genotype 2 Hepatitis E Virus World Health Organization Reference Strain

**DOI:** 10.1128/genomeA.01664-16

**Published:** 2017-02-16

**Authors:** Marco Kaiser, Saleem Kamili, Tonya Hayden, Johannes Blümel, Sally A. Baylis

**Affiliations:** a GenExpress Gesellschaft für Proteindesign mbH, Berlin, Germany; b Division of Viral Hepatitis, Centers for Disease Control and Prevention, Atlanta, Georgia, USA; c Paul-Ehrlich-Institut, Langen, Germany

## Abstract

We report here the sequence of a genotype 2a reference strain of hepatitis E virus (HEV), developed on behalf of the World Health Organization. The HEV reference strain is intended for use in assays based on nucleic acid amplification for the validation of HEV RNA detection.

## GENOME ANNOUNCEMENT

Hepatitis E virus (HEV) is a major cause of acute hepatitis. In humans, infections are caused by four main genotypes (1, 2). These comprise genotypes 1 and 2, which are restricted only to humans, as well as genotypes 3 and 4, which are zoonotic infections of pigs, wild boar, deer, and other animals that can also infect human hosts, typically by the consumption of infected meat and meat products. Recently, HEV genotype 7 was isolated in a patient who regularly consumed camel meat and milk (3). HEV genotypes 1 and 2 cause large waterborne outbreaks of hepatitis E in areas of poor sanitation through transmission of the virus by the fecal–oral route, with most outbreaks caused by HEV genotype 1. In the case of HEV genotype 2, in 1986 there was an outbreak reported in Mexico (4) where one of the virus strains was completely sequenced (5). To date, this remains the only full-length HEV genotype 2 strain reported and it has been designated HEV genotype 2a. Since that time, partial HEV genotype 2 sequences have been identified in hepatitis E outbreaks and sporadic cases in different African countries, including Nigeria (6), Namibia (7), Chad (8), and the Central African Republic (9).

The 1st World Health Organization (WHO) International Reference Panel (IRP, code no. 8578/13) for HEV (10) contains strains representative of one to four HEV genotypes, including genotype 2a. The IRP is intended for standardization of nucleic acid tests, similar to panels developed for other bloodborne viruses (11).

The genotype 2a strain (Mex-14) in the WHO IPR was obtained from a patient in Telixtec, Mexico, in August 1986. For sequence analysis, RNA was purified from the lyophilized HEV IRP sample 8577/13s (prepared from the original stool sample) using the QIAamp MinElute virus spin kit (Qiagen GmbH, Hilden, Germany). Virus RNA was reverse-transcribed using the TaqMan Reverse Transcription Reagents (Thermo Fisher Scientific Life Technologies GmbH, Darmstadt, Germany) with a mixture of random hexamers and specific amplification primers following the manufacturer’s instructions. Published primers (12–14) and additional primers designed according to consensus sequences within known HEV strains and isolate-specific primers were used for the generation of the near full-length sequence. Amplification was performed with Q5 High-Fidelity DNA Polymerase (New England Biolabs, Ipswich, USA) following the manufacturer’s instructions. Amplified products were sequenced directly using the BigDye version 3.1 cycle sequencing kit (Thermo Fisher Scientific Life Technologies GmbH), and products were subjected to an automated sequence analysis using a Genetic Analyser 3500 xl Dx (Thermo Fisher Scientific Inc., USA). Sequence and phylogenetic analyses were conducted using MEGA software version 6.06 (15).

The Mex-14 sequence has a length of 7,159 bp and shares 99% nucleotide identity with M74506, the prototype HEV genotype 2a strain, and represents only the second full-length sequence of an HEV genotype 2 strain; in the context of the WHO IRP, this sequence is an important resource for assay validation and assurance that such strains are adequately detected in nucleic acid amplification assays.

### Accession number(s).

The sequence of the genotype 2 HEV WHO reference strain Mex-14 has been deposited in GenBank under the accession number KX578717.
